# Ferroptosis Characterization in Lung Adenocarcinomas Reveals Prognostic Signature With Immunotherapeutic Implication

**DOI:** 10.3389/fcell.2021.743724

**Published:** 2021-10-20

**Authors:** Sijin Sun, Yannan Yang, Zhenlin Yang, Juhong Wang, Renda Li, He Tian, Fengwei Tan, Qi Xue, Yibo Gao, Jie He

**Affiliations:** ^1^Department of Thoracic Surgery, National Cancer Center, National Clinical Research Center for Cancer, Cancer Hospital, Chinese Academy of Medical Sciences and Peking Union Medical College, Beijing, China; ^2^State Key Laboratory of Molecular Oncology, National Cancer Center, National Clinical Research Center for Cancer, Cancer Hospital, Chinese Academy of Medical Sciences and Peking Union Medical College, Beijing, China

**Keywords:** lung adenocarcinoma, ferroptosis, classification, immunotherapy, machine learning

## Abstract

The iron-dependent cell death named ferroptosis has been implicated in the progression and therapeutic response of several tumors. However, potential role of ferroptosis in lung adenocarcinomas (LUAD) remained less well understood. In TCGA-LUAD cohort, unsupervised clustering was first conducted based on ferroptosis regulators extracted from FerrDb database. Comprehensive correlation analysis and comparisons were performed among ferroptosis subtypes. The ferroptosis-related prognostic (FRP) signature was identified based on filtered features and repeated LASSO and was validated in five independent cohorts. The clinical relevance between the risk score and therapeutic response was further explored by multiple algorithms. qPCR was implemented to verify gene expression. A total of 1,168 LUAD patients and 161 ferroptosis regulators were included in this study. Three ferroptosis subtypes were identified and patients in subtype B had the best prognosis among the three subtypes. Significant differences in immune microenvironment and biological function enrichment were illustrated in distinct subtypes. The Boruta algorithm was conducted on 308 common differentially expressed genes for dimensionality reduction. A total of 56 genes served as input for model construction and a six-gene signature with the highest frequencies of 881 was chosen as FRP. The prognostic significance of FRP was validated in five independent cohorts. High FRP risk score was also linked to increased tumor mutation burden, PD-L1 protein expression and number of neoantigens. Of the FRP genes, 83.3% was abnormally expressed in LUAD cell lines. In conclusion, ferroptosis plays a non-negligible role in LUAD. Exploration of the ferroptosis pattern will enhance the prognostic stratification of individual patients and move toward the purpose of personalized treatment.

## Introduction

Ferroptosis is a non-apoptotic modality of regulated cell death (RCD) that is induced by excessive phospholipid peroxidation that progresses in an iron-dependent manner (Dixon et al., 2012). The biology of ferroptotic cells is morphologically and mechanistically distinct from that of apoptotic cells, as indicated by ferroptosis induction in *BAX/BAK*-deficient cells and the lack of detected caspase-3 cleavage during ferroptosis (Stockwell et al., 2017). Extrinsic and intrinsic pathways that result in unrestricted phospholipid peroxidation can trigger ferroptosis (Tang and Kroemer, 2020). The extrinsic pathway includes blockade of the system x_*c*_^–^ cystine/glutamate antiporter, which is the main transporter of cystine, the rate-limiting precursor of glutathione, and activator of the iron transporters serotransferrin and lactotransferrin, while the intrinsic pathway is initiated by the inhibition of intracellular antioxidant enzymes, such as glutathione peroxidase *GPX4*. Current studies have identified ferroptosis as a critical tumor suppression mechanism, and inhibition of ferroptosis, similar to inhibition of apoptosis, promotes tumor development (Gao et al., 2019; Liu et al., 2019). In addition, multiple tumor suppressors can sensitize cancer cells to ferroptosis, and among these suppressors, p53 has been thoroughly investigated (Zhang et al., 2018, 2019; Jiang et al., 2021). p53 can inhibit the transcription of the systemic x_*c*_^–^ subunit *SLC7A11* (Jiang et al., 2015; Wang et al., 2016), and *P47S* amino acid mutation of p53 was reported to confer resistance to ferroptosis, thus promoting tumor formation (Jennis et al., 2016).

A number of immune checkpoint inhibitors (ICIs) have been developed and utilized in cancer patients in recent decades (He and Xu, 2020), among which anti-PD-1/PD-L1 therapy has led to great success and has been approved to treat a wide variety of cancer types and has led to intermediate response rates (Ribas and Wolchok, 2018). Anti-PD-1/PD-L1 therapy reactivates the immune system to exert antitumor effects by relieving the suppressive functions of PD-1 signaling (Ribas, 2015). Mechanistically, the antitumor effect of ICIs is strongly associated with the host’s immune system and tumor immune microenvironment (TIME). The interaction between cancer cells and the TIME is complicated due to the coexistence of both protumorigenic and antitumorigenic factors. Induction of the T cell-inflamed TIME induced by JHU083 (Leone et al., 2019), CDK4/6 (Schaer et al., 2018), DCR-BCAT (Ganesh et al., 2018), or other treatments has been demonstrated to enhance the efficacy of ICIs. Thus, deeper insight into the TIME is needed to facilitate the identification of novel predictive biomarkers and the development of novel therapeutic strategies.

Recently, several studies have explored the specific association between ferroptosis and TIME infiltrating immune cells. It has been reported that anti-PD-L1 and ferroptosis inducers [such as erastin, *RSL3*, and cyst(e)inase] synergistically inhibit tumor development both *in vivo* and *in vitro* (Wang et al., 2019). Mechanistically, interferon-γ (IFNγ) released from activated cytotoxic T cells downregulates the expression of *SCL7A11* and *SLC3A2* through activation of the JAK-STAT1 pathway and thus induces ferroptosis of cancer cells. However, the cellular components released by ferroptotic cancer cells play dual roles in antitumor immunity. KRAS-G12D within exosomes secreted by ferroptotic pancreatic cancer cells can be absorbed by macrophages through *AGER*, which results in the polarization of macrophages to the M2 phenotype and an immunosuppressive microenvironment for tumor growth (Dai et al., 2020a). In addition, ferroptosis induced by conditional depletion of *Gpx4* in the pancreas promotes mutant Kras-driven tumorigenesis in mice through secretion of DNA and activation of the TMEM173/STING-dependent DNA sensor pathway in macrophages (Dai et al., 2020b).

Although success in revealing the molecular mechanisms between ferroptosis and TIME has been achieved, the abovementioned studies focused on limited ferroptosis regulators and were mainly conducted *in vitro*. Therefore, comprehensive analysis of ferroptosis regulators and TIME in real-world cohorts of patients is urgently needed. In this study, we integrated transcriptome datasets of 500 lung adenocarcinoma (LUAD) samples from The Cancer Genome Atlas (TCGA) and found three distinct ferroptosis subtypes, among which significant survival differences, diverse biological pathways, and different infiltrated immune cells were observed. Furthermore, we constructed a robust ferroptosis-related prognostic (FRP) signature composed of 6 genes (*EIF5A*, *CACYBP*, *CYCS*, *ANLN*, *ARNTL2*, and*PPM1M*) based on defined ferroptosis subtypes and confirmed the FRP signature as an independent prognostic factor for LUAD patients. Notably, patients in the high-risk FRP signature group may potentially benefit from immunotherapy due to the elevation of tumor mutation burden (TMB) and PD-L1 and neoantigen expression. In conclusion, our results revealed that ferroptosis plays critical roles in LUAD and that the FRP signature may be a promising predictive biomarker for prognosis and immunotherapy response.

## Materials and Methods

### Lung Adenocarcinoma Dataset Acquisition and Preprocessing

Public lung adenocarcinoma transcriptome datasets were obtained from The Cancer Genome Atlas (TCGA) and Gene Expression Omnibus (GEO). Patients without prognostic information were excluded from the study. A total of six datasets (TCGA-LUAD, GSE13213, GSE30219, GSE31210, GSE3141, and GSE41271) were gathered for further analysis (Bild et al., 2006; Tomida et al., 2009; Okayama et al., 2012; Rousseaux et al., 2013; Sato et al., 2013). For RNA-Seq data from the TCGA, FPKM values were downloaded from the TCGA Data Portal^1^ and transformed into TPM values before analysis. For datasets in the GEO, normalized probe expression values generated from the microarray were acquired using the R package GEOquery (Davis and Meltzer, 2007). The probes in each dataset were annotated according to the appropriate platform annotation file. When multiple probes mapped to the same gene, the average expression values of these genes were used as the final expression value. To calculate tumor mutation burden (TMB) in TCGA-LUAD, gene-level mutation (VarScan) data were extracted from the TCGA Data Portal. In the present study, TMB was defined as the total number of non-synonymous mutations divided by the size of the exome.

### Consensus Clustering of Ferroptosis Regulators

A comprehensive list of ferroptosis drivers and suppressors was acquired from the FerrDb database^2^ (Zhou and Bao, 2020). After removing duplicated entries and performing univariate Cox regression for each gene, 37 unique prognosis-associated genes were obtained for unsupervised clustering. The identification of ferroptosis subtypes was conducted with the TCGA cohort by applying the partitioning around medoids cluster algorithm in the R package ConsensusClusterPlus (Wilkerson and Hayes, 2010). The number of repetitions was set to 100 to ensure the stability of the result. The other parameters of the cluster algorithm were set to the default values.

### Gene Set Variation Analysis and Functional Comparison Between Subtypes

To determine the biological function associated with different ferroptosis subtypes, GSVA was performed to detect subtle pathway activity in each sample using the R package GSVA (Hänzelmann et al., 2013). GSVA is a non-parametric, unsupervised method for calculating gene set enrichment through expression profiles. The gene set file named “c2.cp.kegg.v7.4.symbols.gmt,” including 186 canonical pathways, was downloaded from MSigDB and was chosen for pathway enrichment (Liberzon et al., 2011). A pathway comparison was conducted using the R package limma (Smyth, 2005), and an adjusted *p* value of less than 0.05 was considered statistically significant.

### Exploration of Tumor-Infiltrating Immune Cells in the TCGA-Lung Adenocarcinomas Cohort

Two widely validated algorithms, CIBERSORT and ImmuCellAI (Newman et al., 2015; Miao et al., 2020), were implemented to estimate the abundance of specific immune components. CIBERSORT is a computational approach reinforced by support vector regression to quantify 22 immune cell fractions. ImmuCellAI was used for abundance estimation of 24 immune cell types, including 18 T cell subsets, from gene expression matrices. The absolute fraction and abundance of immune cells were estimated by CIBERSORT and ImmuCellAI, respectively. Only intergroup comparisons were conducted and the differences in immune cell abundance were depicted between risk groups and among subtypes.

### Construction of a Ferroptosis-Related Prognostic Model

To identify common DEGs among the three ferroptosis subtypes, gene expression was quantified by TPM value and empirical Bayes statistics was applied for differential expression analysis using limma package (Smyth, 2005). The obtained *p-*values were further adjusted by the Benjamini–Hochberg method. The DEGs were defined as genes with an adjusted *p*-value less than 0.001. By taking the intersection of the outputs, common DEGs of the three subtypes were obtained and illustrated using a Venn diagram. Next, the Boruta feature selection algorithm was applied to narrow the genes for model construction using the R package Boruta (Kursa and Rudnicki, 2010). Principal component analysis (PCA) was p to verify the ability to distinguish subtypes using selected genes. Furthermore, genes related to prognosis were screened out through univariate Cox regression and served as input for model training. To generate a robust ferroptosis-related prognostic (FRP) signature, LASSO Cox regression with 10-fold cross-validation was performed using a random seed, and the whole procedure was repeated 1,000 times (Tibshirani, 1997). The model with the highest frequency was selected as the final signature. Patients were subsequently stratified into high-risk and low-risk groups according to the median of the risk score. Both Kaplan–Meier curves and multivariate Cox regression were performed in the training cohort.

### Evaluation of Ferroptosis Activity in the Different Risk Groups

To quantify the activity of ferroptosis, an established indicator named the ferroptosis potential index (FPI) was obtained from a previous study and calculated for each sample based on gene expression data (Liu et al., 2020). During computation, key ferroptosis regulator genes were first divided into positive and negative groups according to their potential function. Then, the enrichment score of these two groups was calculated separately using the R package GSVA (Hänzelmann et al., 2013). The FPI was defined as the enrichment score of the positive group minus the negative group and was considered a reliable indicator of ferroptosis level.

### Validation of the Ferroptosis-Related Prognostic Signature in Multiple Validation Cohorts

To further validate the prognostic value of the FRP signature, the risk score was first calculated for each sample based on the formula derived from the training cohort. Then, samples were divided into low-risk and high-risk groups according to the median risk score in each cohort. Kaplan–Meier curves were generated, and univariate Cox regression was performed, and the concordance index (C-index) was applied to evaluate the performance of the FRP signature.

### Exploring the Implications of the Ferroptosis-Related Prognostic Signature on Immunotherapeutic and Chemotherapeutic Responses

To infer the potential benefit of immunotherapy for each patient, the number of neoantigens and the PD-L1 protein expression of patients in the TCGA-LUAD cohort were extracted from The Cancer Immunome Atlas^3^ and cBioPortal for Cancer Genomics^4^, respectively (Gao et al., 2013; Charoentong et al., 2017). To determine the chemotherapeutic implications, experimental data for cancer cell drug sensitivity were obtained from the Genomics of Drug Sensitivity in Cancer (GDSC), and the whole prediction process was conducted with the R package pRRophetic (Geeleher et al., 2014). “allSoldTumours” was selected for the tissue type parameter, and the remaining parameters were set to the default values.

### Cell Lines and Cell Culture Conditions

BEAS-2B and HCC827 cell lines were purchased from ATCC. BEAS-2B cells were cultured in BEBM basal medium supplemented with 10% fetal bovine serum (FBS), 1,000 U ml^–1^ penicillin and 100 μg ml^–1^ streptomycin. HCC827 cells were maintained in RPMI 1640 supplemented with 10% FBS, 1,000 U ml^–1^ penicillin and 100 μg ml^–1^ streptomycin. The cells were cultured with 5% CO_2_ at 37°C in a humidified incubator.

### RNA Extraction and Quantitative RT-PCR Analysis

Total RNA was extracted from cells using TRIzol reagent (Invitrogen, United States) according to the manufacturer’s instructions. Equal amounts of RNA samples were used for cDNA synthesis with a RevertAid First Strand cDNA synthesis kit (Thermo). SYBR Green-based qRT-PCR on a 7900HT fast real-time PCR system (Applied Biosystems/Life Technologies, Waltham, United States) was performed. The relative mRNA expression levels were calculated by the 2^–ΔΔ^*^*C*^*^*t*^ method with normalization to *ACTB*; the PCR primers are listed in Supplementary Table 1.

### Statistical Analysis

All bioinformatic analyses were conducted in R software (Version 3.6.3). Wilcoxon test and Kruskal–Wallis test were performed to statistically test the difference in continuous data for defined subtypes. The Spearman coefficient was used to evaluate the association between variables. Survival analyses, including Kaplan–Meier curves and Cox regression, were performed using the R package survival. The C-index was calculated by R package survcomp. A *P*-value less than 0.05 was considered statistically significant.

## Results

### Identification of Distinct Ferroptosis Subtypes on the Basis of Ferroptosis Regulators

**FIGURE 1:**
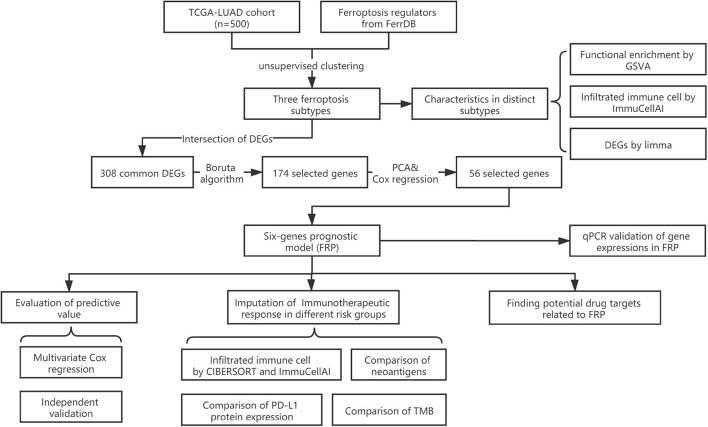
Flowchart of the present study.

**FIGURE 2:**
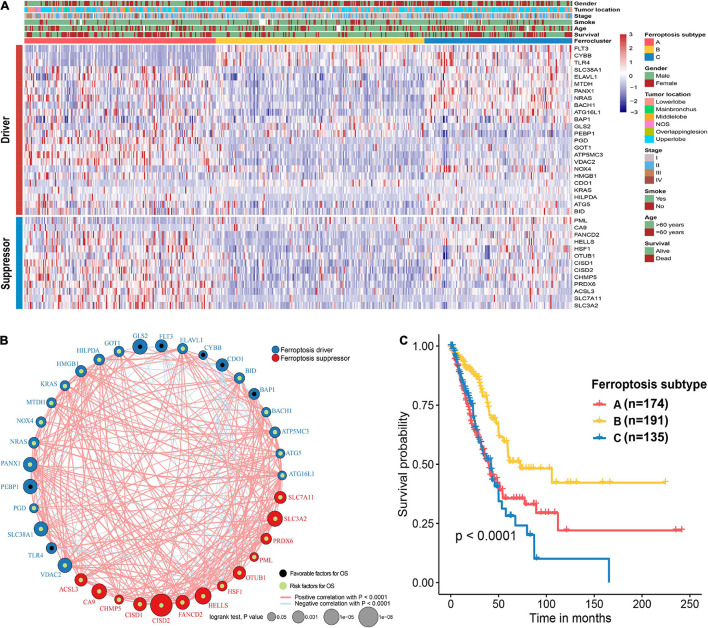
Unsupervised clustering of ferroptosis regulators and characteristics of ferroptosis subtypes. **(A)** Heat map showing the expression patterns of 37 ferroptosis regulator genes in the TCGA-LUAD cohort. Sex, tumor location, stage, smoking history, age, and survival status were the annotations. **(B)** Interactions of 37 ferroptosis regulator genes. The size of each circle indicates the survival significance of each gene. Ferroptosis drivers are shown in blue, and ferroptosis suppressors are shown in red. Favorable prognostic factors are indicated in black, and unfavorable prognostic factors are indicated in green. The strength of the relationship between two genes is symbolized by the thickness of the line. **(C)** Kaplan–Meier curves of the overall survival in the TCGA-LUAD cohort on the basis of the ferroptosis subtypes.

The flow chart of this study is shown in Figure 1. A comprehensive list of genes including 108 that encode ferroptosis drivers and 69 that encode suppressors was extracted from the FerrDb. After removing multirole regulators that would be repeatedly counted, a total of 161 genes remained for screening. Univariate Cox regression was performed, and 37 genes with prognostic significance (*p* < 0.05) were chosen from the list. Based on the expression level of these genes, three subtypes were identified in the TCGA-LUAD cohort. The clinical information and gene expression patterns are displayed in Figure 1. In subtype A, most of the genes, including both drivers and suppressors, were highly expressed, and in contrast, a substantial portion of ferroptosis regulators was expressed at low levels in subtype B (Figure 2A). The expression pattern in subtype C was somewhere in between the other two subtypes. An association analysis suggested that there was a close connection between the expression levels of the 37 ferroptosis regulators (Figure 2B). Of 24 ferroptosis drivers, nearly one-third (7/24) of the genes was revealed to be favorable prognostic factors, and the remaining genes (17/24) were risk factors for overall survival (OS; Figure 2B). All 13 ferroptosis suppressors were unfavorable prognostic factors (Figure 2B). These results suggested that inhibition of ferroptosis suppressors may improve the survival of LUAD patients. In addition, a significant survival difference (*p* < 0.0001) was observed among the three ferroptosis subtypes (Figure 2C). Patients with subtype B had the best prognosis compared with patients with the other subtypes, while those with subtype C had the worst prognosis (Figure 2C). The clinicopathological characteristics of the three subtypes were summarized in Table 1.

**TABLE 1:** Correlations between ferroptosis subtypes and clinicopathological parameters in TCGA-LUAD cohort.

Category	Ferroptosis subtypes		
	Subtype A (*n* = 163)	Subtype B (*n* = 174)	Subtype C (*n* = 127)
**Age [mean (*SD*)]**	63.3 (10.3)	67.0 (9.6)	65.3 (9.6)
**Gender**			
Male	97(59.5%)	58(33.3%)	56(44.1%)
Female	66(40.5%)	116(66.7%)	71(55.9%)
**Smoking**			
Yes	147(90.2%)	142(81.6%)	110(86.6%)
No	16(9.8%)	32(18.4%)	17(13.4%)
**T stage**			
T1	38(23.3%)	87(50.0%)	36(28.3%)
T2	96(58.9%)	71(40.8%)	77(60.6%)
T3	22(13.5%)	13(7.5%)	9(7.1%)
T4	7(4.3%)	3(1.7%)	5(3.9%)
**N stage**			
N0	99(60.7%)	140(80.5%)	74(58.3%)
N1	37(22.7%)	20(11.5%)	27(21.3%)
N2	27(16.6%)	14(8.0%)	24(18.9%)
N3	0(0.0%)	0(0.0%)	2(1.6%)
**M stage**			
M0	150(92.0%)	171(98.3%)	122(96.1%)
M1	13(8.0%)	3(1.7%)	5(3.9%)
**TNM stage**			
I	75(46.0%)	121(69.5%)	60(47.2%)
II	44(27.0%)	34(19.5%)	34(26.8%)
III	31(19.0%)	16(9.2%)	28(22.0%)
IV	13(8.0%)	3(1.7%)	5(3.9%)
**Status**			
Survival	87(53.4%)	137(78.7%)	75(59.1%)
Death	76(46.6%)	37(21.3%)	52(40.9%)
**Tumor location**			
Lower lobe	53(32.5%)	65(37.4%)	36(28.3%)
Middle lobe	8(4.9%)	7(4.0%)	5(3.9%)
Upper lobe	90(55.2%)	98(56.3%)	84(66.1%)
Main bronchus	2(1.2%)	0(0.0%)	0(0.0%)
Overlapping lesion	2(1.2%)	2(1.1%)	0(0.0%)
Lung NOS	8(4.9%)	2(1.1%)	2(1.6%)

### Functional Enrichment and Immune Microenvironment Assessment of the Ferroptosis Subtypes

**FIGURE 3:**
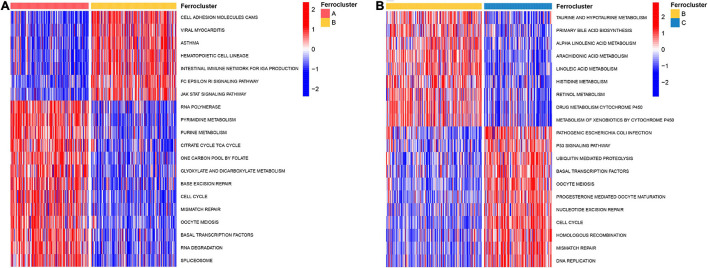
Biological characteristics of different ferroptosis subtypes. GSVA analysis revealed highly enriched KEGG pathways in distinct ferroptosis subtypes. The 20 most enriched pathways as identified in each comparison are visualized by heat map. **(A)** Ferroptosis subtype A vs. subtype B. **(B)** Ferroptosis subtype B vs. subtype C.

**FIGURE 4:**
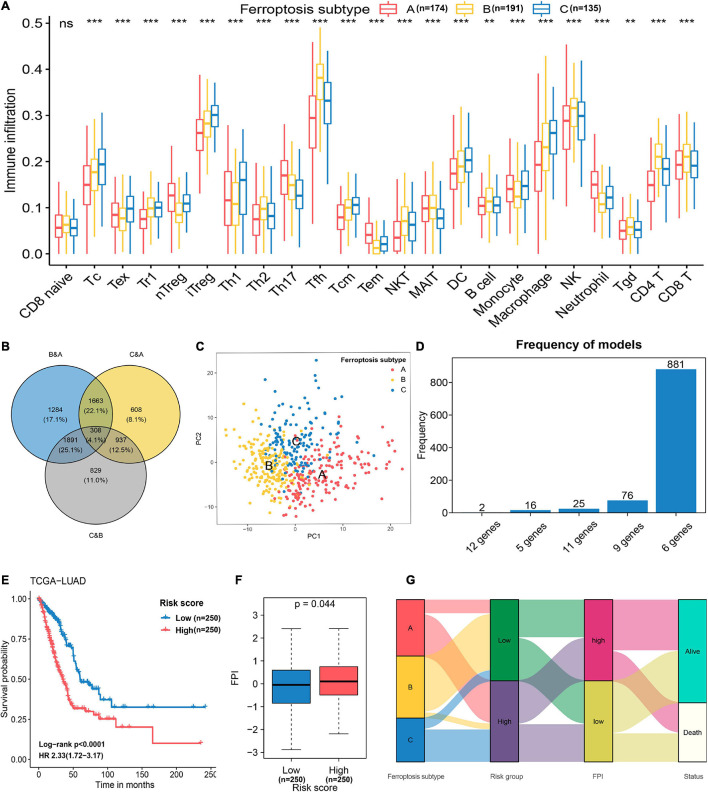
Feature selection and signature construction process. **(A)** Assessing the abundance of infiltrated immune cells in distinct ferroptosis subtypes. **p < 0.01, ***p < 0.001; ns, non-significant. **(B)** The number of unique and shared DEGs from different comparisons as revealed in a Venn diagram. **(C)** Principal component analysis of 174 genes selected with the Boruta feature selection algorithm. These genes can effectively discriminate the different ferroptosis subtypes. **(D)** The distribution of model occurrences after 1,000 iterations of LASSO regression. The six-gene prognostic signature was selected because it had a higher frequency than the other four models. **(E)** Kaplan–Meier curves and univariate Cox regression revealed that the FRP risk score was markedly related to overall survival in the TCGA-LUAD cohort. **(F)** Differences in FPI scores between the high-risk and low-risk groups. **(G)** Alluvial diagram showing the relations of ferroptosis subtype, FRP risk group, FPI, and status.

To further explore the biological pathway underlying each distinct ferroptosis subtype, GSVA was performed to determine pathway enrichment (Subtype A vs. Subtype B; Subtype B vs. Subtype C). As shown in Figure 3A, ferroptosis subtype A was significantly enriched in genes involved with RNA polymerase, pyrimidine metabolism, purine metabolism, cell cycle, and mismatch repair. Ferroptosis subtype B was markedly enriched in genes related to fatty acid and drug metabolism, including bile acid biosynthesis, alpha-linolenic acid metabolism, arachidonic acid metabolism, linoleic acid metabolism, and cytochrome P450 drug metabolism (Figure 3B). In addition, subtype B genes are also involved in cell adhesion and molecules (CAMS), intestinal immune network, and JAK-STAT signaling pathway (Figure 3A). Subtype C genes were predominantly related to pathogenic *Escherichia coli* infection, the p53 signaling pathway, ubiquitin-mediated proteolysis, and basal transcription factors (Figure 3B). Subsequent evaluation of infiltrated immune cells revealed conspicuous differences in the immune microenvironment among subtypes (Figure 4A). Particularly, as the subtype with the best prognosis, the ferroptosis subtype B genes were prominently enriched in Tfh, NKT, NK, CD4^+^ T, and CD8^+^ T cells (Figure 4A).

### Construction of an Ferroptosis-Related Prognostic Signature for the TCGA-Lung Adenocarcinomas Cohort

To construct a prognostic model related to the defined ferroptosis subtypes, the intersecting DEGs identified in the pairwise comparisons were identified, and a total of 308 genes were selected as initial features (Figure 4B). After the dimension reduction by the Boruta algorithm, PCA was performed on the residual 174 genes, and the results indicated that the expression of these genes had great discriminatory power for the different subtypes (Figure 4C). Thus, these 174 genes constituted a representative collection of genes in the defined ferroptosis subtypes. These demonstrated that the dimensionality reduction process successfully removed unuseful features (*n* = 134) and retained relevant genes (*n* = 174). By applying univariate Cox regression, 56 genes with prognostic significance (*p* < 0.05) were retained for repeated LASSO Cox regression to generate the best model (Supplementary Table 2). A total of 1,000 iterations were conducted, and the distribution of model occurrences is shown in Figure 4D. As presented in Figure 4D, a prognostic signature with 6 genes (*EIF5A*, *CACYBP*, *CYCS*, *ANLN*, *ARNTL2*, and *PPM1M*) had the highest frequencies, with 881, which greatly exceeded those of the other four models and was, therefore, chosen as the FRP signature. The risk score of the FRP signature was calculated as follows: risk score = (0.0413 × EXP*_*EIF5A*_*) + (0.0068 × EXP*_*CACYBP*_*) + (0.0254 × EXP*_*CYCS*_*) + (0.1300 × EXP*_*ANLN*_*) + (0.1503 × EXP*_*ARNTL2*_*) – (0.0848 × EXP*_*PPM1M*_*). After calculating the risk score for each sample in the training cohort, samples were stratified into low-risk and high-risk groups based on the median risk score, and the survival analysis demonstrated that patients in the high-risk group had a significantly shorter overall survival time (log-rank *p* < 0.001, HR = 2.33, 95% CI = 1.72–3.17) than patients in the low-risk group (Figure 4E). The FPI was applied to quantify the activity of ferroptosis in each group, and the results suggested that the FPI in the high-risk group was significantly (*p* = 0.044) higher than that in the low-risk group (Figure 4F), which indicated a higher ferroptosis activity in samples that with higher risk. The association of attributes including ferroptosis subtype, risk group, FPI, and survival status of individual patients is presented in an alluvial diagram (Figure 4G).

### Identification of the Ferroptosis-Related Prognostic Signature as an Independent Prognostic Factor

**FIGURE 5:**
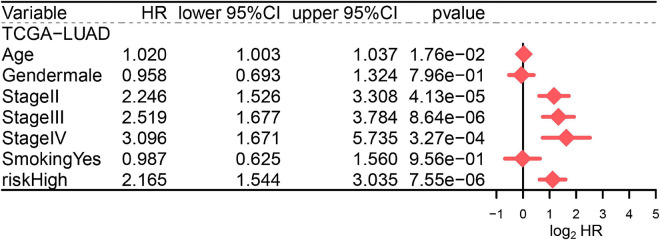
Multivariate Cox regression analysis including age, sex, stage, smoking history, and FRP risk score in the TCGA-LUAD cohort.

To decrease the effects of other confounding factors on the relationship between the FRP risk score and OS time, multivariate Cox regression analysis including age, sex, stage, and smoking history was conducted with the TCGA-LUAD cohort. The results proved that age, stage, and risk score (*p* < 0.001, HR = 2.17, 95% CI = 1.54–3.04) were independent prognostic factors of the OS time for LUAD patients (Figure 5).

### Validation and Evaluation of the Predictive Performance of the Ferroptosis-Related Prognostic Signature in Five Validation Cohorts

**FIGURE 6:**
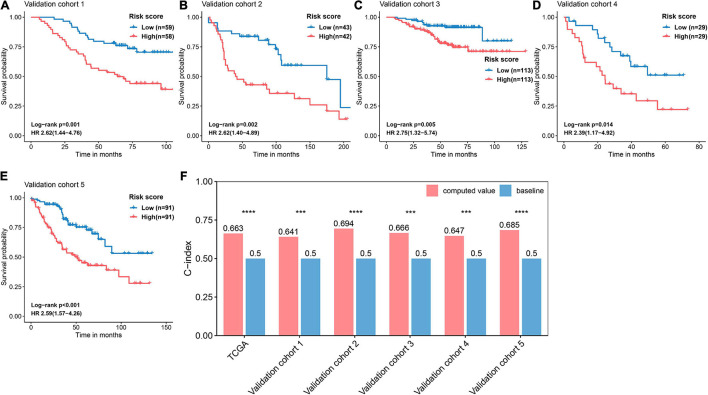
Validation and evaluation of the performance of the FRP risk score. Kaplan–Meier curves and univariate Cox regression were performed with respect to the different risk groups in **(A)** validation cohort 1 (GSE13213), **(B)** validation cohort 2 (GSE30219), **(C)** validation cohort 3 (GSE31210), **(D)** validation cohort 4 (GSE3141), and **(E)** validation cohort 5 (GSE41271). **(F)** Assessing the predictive performance of the FRS by the C-index. The C-index was calculated for each cohort and compared to the null hypothesis. ***p < 0.001, ****p < 0.0001.

The basic information related to the five validation cohorts is described in Table 2. Briefly, a total of 668 cases made up our validation datasets. Consistent and significant differences were observed in validation cohort 1 (log-rank *p* = 0.001, HR = 2.62, 95% CI = 1.44–4.76), validation cohort 2 (log-rank *p* = 0.002, HR = 2.62, 95% CI = 1.40–4.89), validation cohort 3 (log-rank *p* = 0.005, HR = 2.75, 95% CI = 1.32–5.74), validation cohort 4 (log-rank *p* = 0.014, HR = 2.39, 95% CI = 1.17–4.92), and validation cohort 5 (log-rank *p* < 0.001, HR = 2.59, 95% CI = 1.57–4.26; Figures 6A–E). In addition, the C-index was calculated to assess predictive performance and the C-index was 0.663 for the training cohort, 0.641 for validation cohort 1, 0.694 for validation cohort 2, 0.666 for validation cohort 3, 0.647 for validation cohort 4, and 0.685 for validation cohort 5 (Figure 6F). Statistical significance (*p* < 0.05) was observed between the actual value of the C-index and the null hypothesis (C-index = 0.5) in all datasets (Figure 6F).

**TABLE 2:** Description of the training and validation cohorts used in this study.

Dataset	Source	Accession	Platform	Number of samples
Training cohort	TCGA	TCGA-LUAD	Illumina HiSeq	500
Validation cohort 1	GEO	GSE13213	GPL6480	117
Validation cohort 2	GEO	GSE30219	GPL570	85
Validation cohort 3	GEO	GSE31210	GPL570	226
Validation cohort 4	GEO	GSE3141	GPL570	58
Validation cohort 5	GEO	GSE41271	GPL6884	182

### Depiction of the Immune Microenvironment and Implications of the Immunotherapeutic Response Between Risk Groups

**FIGURE 7:**
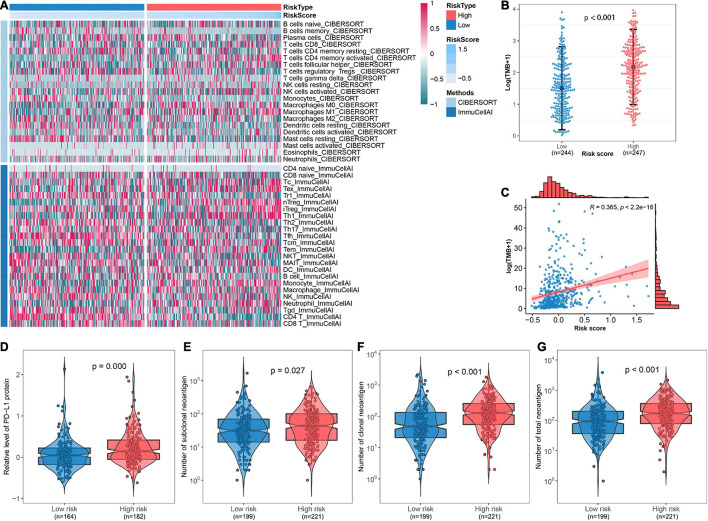
Depiction of the immune microenvironment and implications of the immunotherapeutic response. **(A)** Heat map showing the immune microenvironment of the TCGA-LUAD cohort as estimated by CIBERSORT and ImmuCellAI. **(B)** Comparison of TMB between high-risk and low-risk groups. **(C)** Scatter plot presenting the correlation between TMB and risk score. Comparisons of **(D)** the expression of PD-L1 protein, **(E)** number of subclonal neoantigens, **(F)** number of clonal neoantigens, and **(G)** number of total neoantigens between different risk groups.

The abundance of infiltrated immune cells was estimated using CIBERSORT and ImmuCellAI for each sample, and a heat map was generated to illustrate the association between immune cell infiltration and the FRP risk score (Figure 7A). The top five immune cell types with significant changes in abundance between risk groups were CD4^+^ T cells (*p* < 0.001), nTregs (*p* < 0.001), Tfhs (*p* < 0.001), Tems (*p* < 0.001), and monocytes (*p* < 0.001). The detailed results of these differential analyses are presented in Supplementary Table 3. Furthermore, an association analysis of the FRP risk score and potential immunotherapeutic biomarkers, including TMB, PD-L1 protein, and neoantigen, was performed. The results suggested that TMB in the high-risk group was significantly (*p* < 0.001) higher than that in the low-risk group (Figure 7B). A correlation analysis revealed that the FRP risk score was significantly associated (*R* = 0.365, *p* < 0.0001) with TMB (Figure 7C). In addition, the expression level of the PD-L1 protein (*p* < 0.001) and the numbers of subclonal neoantigens (*p* = 0.027), clonal neoantigens (*p* < 0.001), and total neoantigens (*p* < 0.001) were all significantly higher in the high-risk group than in the low-risk group (Figures 7D–G). These results implied that patients in the high-risk group may potentially benefit from immunotherapy.

### Extrapolating the Relationship Between the Ferroptosis-Related Prognostic Signature and Chemotherapy Response

**FIGURE 8:**
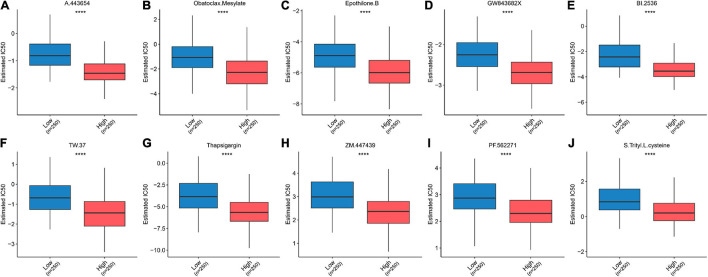
Box plots based on the estimated IC50 of the 10 most significant compounds: **(A)** A.443654, **(B)** obatoclax mesylate, **(C)** epothilone B, **(D)** GW843682X, **(E)** BI.2536, **(F)** TW.37, **(G)** thapsigargin, **(H)** ZM.447439, **(I)** PF.562271, and **(J)** *S*-Trityl-L-cysteine. ****p < 0.0001.

Considering that chemotherapy constitutes an important component of LAUD treatment, drug susceptibility data including 138 compounds from the GDSC were applied to the training group to predict drug sensitivities for each sample in the TCGA-LUAD cohort. Based on the significance level, the top 10 compounds that might benefit patients in the high-risk group were identified: A.443654, obatoclax mesylate, epothilone B, GW843682X, BI.2536, TW.37, thapsigargin, ZM.447439, PF.562271, and *S*-Trityl-L-cysteine (Figures 8A–J). These results provide a novel perspective for exploring new drug candidates. A detailed list showing the significance value for all 138 compounds is available in Supplementary Table 4.

### Quantification of Gene Expression in Normal and Lung Adenocarcinoma Cell Lines

**FIGURE 9:**
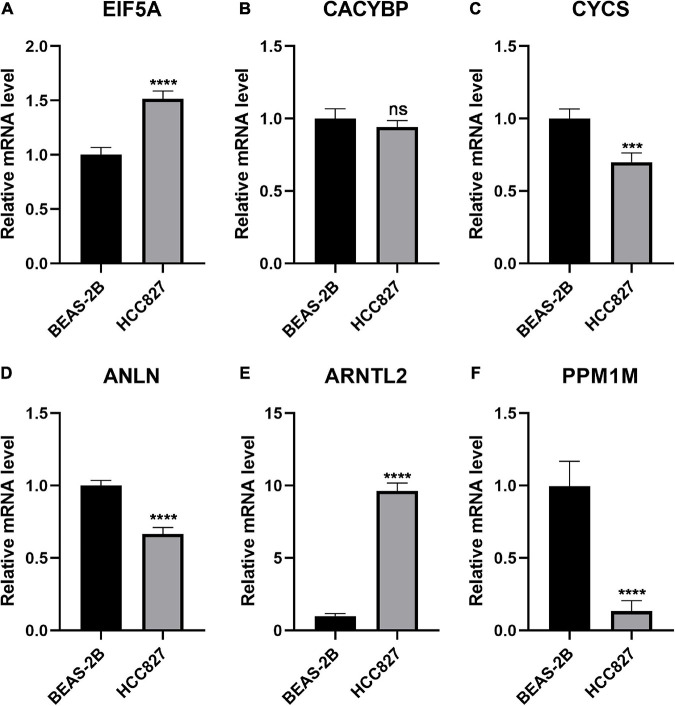
Exploration of the expression of the six genes in normal and LUAD cell lines. Quantification and comparison of mRNA expression in the FRP signature as determined by qPCR: **(A)** EIF5A, **(B)** CACYBP, **(C)** CYCS, **(D)** ANLN, **(E)** ARNTL2, and **(F)** PPM1M. ***p < 0.001, ****p < 0.0001; ns, non-significant.

To further investigate the expression pattern *in vitro*, six genes that constituted the FRP signature were quantified by qPCR and those in normal bronchial epithelial cells (BEAS-2B cells) and LUAD cells (HCC827 cells) were compared. Of these genes, *EIF5A* and *ARNTL2* were significantly overexpressed in tumor cells (*p* < 0.05; Figures 9A,E). In contrast, the expression levels of *CYCS*, *ANLN*, and *PPM1M* were significantly elevated in normal lung cells (*p* < 0.05; Figures 9C,D,F). No significant difference in *CACYBP* expression was observed between the two cell lines (Figure 9B).

## Discussion

Increasing studies about the association between ferroptosis and the TIME have been performed since the term ferroptosis was coined in 2012, and most of these studies demonstrated that ferroptosis might contribute to the immune surveillance and cytotoxic effects of CD8^+^ T cells (Wang et al., 2019), while others reported that ferroptosis facilitated the formation of a tumor-promoting immune microenvironment (Wen et al., 2019). As the roles of ferroptosis in shaping the tumor suppressive or promoting microenvironment remain elusive and most studies focus on a single ferroptotic regulator and a single cell type populating the TIME, the overall characteristics of the TIME linked to a specific ferroptotic signature in LUAD need to be elaborated.

Here, we identified three distinct ferroptosis subtypes based on the expression level of 37 ferroptosis driver and suppressor genes with prognostic significance (*p* < 0.05) in the TCGA-LUAD cohort. Patients with ferroptosis subtype B, in which most of the ferroptosis regulators were expressed at low levels, had the best prognosis. In addition, we evaluated the infiltrated immune cells in these three ferroptosis subtypes and found that subtype B was significantly enriched in Tfh, NKT, NK, CD4^+^ T, and CD8^+^ T cells, compared with the other two subtypes, suggesting that the TIME in association with ferroptosis subtype B was enriched in tumor-suppressive immune cells, which can partially explain the reason that patients with subtype B have the best prognosis.

Then, we constructed an FRP signature consisting of 6 genes (*EIF5A*, *CACYBP*, *CYCS*, *ANLN*, *ARNTL2*, and *PPM1M*) based on the defined ferroptosis subtypes and identified the FRP signature as an independent prognostic factor (*p* < 0.001, HR = 2.17, 95% CI = 1.54–3.04) in the training TCGA-LUAD cohort and five validation cohorts. Patients in the high-risk group had a significantly shorter overall survival time than patients in the low-risk group. In addition, we applied the FPI to quantify the overall activity of ferroptosis in each group and unsurprisingly found that the FPI in the low-risk group patients, who had a better prognosis, was significantly lower than that in the high-risk group, which was in accordance with the abovementioned results indicating that most of the ferroptosis regulators are expressed at low levels in patients with ferroptosis subtype B, who have the best prognosis, and a previous study showing that a high FPI predicts a poor prognosis (Liu et al., 2020).

Among the six genes in the FRP signature, *ANLN* and *ARNTL2* were mechanistically explored in LUAD in previous studies. *ANLN*, a homolog of anillin whose stability and nuclear localization are regulated by the PI3K/AKT pathway, can bind to and activate *RHOA*, thus promoting cancer growth and development (Suzuki et al., 2005). In addition, Xu et al. (2019) reported that *ANLN* is involved in the metastasis of LUAD by promoting epithelial mesenchymal transformation of tumor cells, and Long et al. (2018) pointed out that higher expression of *ANLN* predicted a relatively poor prognosis. *ARNTL2* is a transcription factor that is involved in promoting LUAD metastasis by orchestrating the expression of a complex prometastatic secretome (Brady et al., 2016). With respect to the other genes, *EIF5A*, a eukaryotic translation initiation factor, enhances the expression of a series of proliferation-associated proteins essential for cancer cells and is upregulated in several cancers, including pancreatic ductal adenocarcinoma, glioblastoma, leukemia, liver, colon, lung, cervical, and ovarian cancer (Mathews and Hershey, 2015; Strnadel et al., 2017). *CACYBP*, a calcyclin-binding protein, interacts with different partner proteins and, through these interactions, is involved in various cellular processes, such as proliferation, differentiation, cytoskeletal reorganization, and protein ubiquitination (Lian et al., 2019). For example, the Siah1-interacting protein (SIP)/CACYBP complex participates in the ubiquitination and proteasomal degradation of β-catenin, an oncogenic protein that regulates gene transcription (Jiang et al., 2019). Previous studies have reported that *CACYBP* contributes to the development of a wide range of human malignancies, including gastric, pancreatic, colon, breast, brain, and renal cancer (Topolska-Wos et al., 2016). *CYCS*, also known as cytochrome c, is a small soluble heme protein that shuttles electrons during the oxidative phosphorylation process and engages in the intrinsic apoptotic pathway under proapoptotic conditions (Ow et al., 2008). *PPM1M*, protein phosphatase 1M, is a manganese/magnesium ion-dependent serine/threonine phosphatase (Kamada et al., 2020). It was reported that *PPM1M* inhibits IL-1-induced activation of NF-κB by dephosphorylating IKK β (Henmi et al., 2009).

We also determined the abundance of infiltrated immune cell types in different risk groups using CIBERSORT and ImmuCellAI. The low-risk group with a better prognosis was enriched with CD4^+^ T cells and Tfhs, which was consistent with the infiltrating cell types associated with ferroptosis subtype B, and the abundance of nTregs, Tems, and monocytes was relatively low. Follicular helper T (Tfh) cells, emerging as a fifth helper T subset, interact with B cells, driving their differentiation into long-lived antibody-secreting plasma cells or memory B cells (King et al., 2008). In breast cancers, Tfh cells recruit immune cells to the TIME and promote tertiary lymphoid structure formation, in which effective antitumor immune responses can be generated and maintained (Gu-Trantien et al., 2013). Certain TIMEs were affected by several factors including exercise, age, diet, adiposity, the microbiome, sex, and tumor-dependent effects. Comprehensive consideration of the above factors during research will be more conducive to dissect the formation mechanism of TIME and lay the foundation for developing intervention measures (Binnewies et al., 2018). Our results suggested that patients in the low-risk group may have a better prognosis partially because of the infiltration of Tfh cells, which facilitate the formation of an antitumor TIME. Furthermore, we found that potential immunotherapeutic biomarkers, including TMB, the PD-L1 protein, and neoantigens, were elevated in the high-risk group, suggesting that patients in the high-risk group may benefit the most from immunotherapy. Our results revealed distinct immune and mutational profiles between different risk groups. Thus, we recommend to include ferroptosis-related genes or ferroptosis signature in future immunotherapy cohort for biomarker study as compared with previous ferroptosis associated prognostic model (Wang et al., 2021), which was directly constructed utilizing LASSO Cox regression model in TCGA-LUAD dataset and validated in one GEO dataset. By contrast, we firstly used ferroptosis regulators to identify three ferroptosis subtypes with prognostic significance and constructed a FRP signature by repeated LASSO Cox using 308 subtype-distinguishing genes. We also validated the signature in five GEO datasets and evaluated its implications of the immunotherapeutic response and chemotherapy, which was of great importance for further clinical application.

Inevitably, several limitations should be acknowledged in our study: (1) A total of 6 cohorts (a TCGA-LUAD cohort and 5 GEO cohorts) used for the construction and further validation of the FRP signature were downloaded from public databases retrospectively. A study with a prospective clinical trial cohort is needed to verify the clinical application value of our FRP signature. (2) Ferroptosis regulators obtained from the FerrDb website were manually extracted from previous literature reports, and therefore, some unselected crucial ferroptosis-associated genes may be missing in our study. In addition, ferroptosis regulators have been reported to regulate ferroptosis in human cells but not specifically in LUAD. (3) The FRP signature in our study was constructed based on DEGs identified in three ferroptosis subtypes, and the six genes in the FRP signature have not yet been identified to be involved in ferroptosis. Further studies are needed to determine the association and mechanisms between the six FRP genes and ferroptosis in LUAD. (4) Algorithmic calculation of immune cell abundance may not be as accurate as immunofluorescence, and further verification is required.

## Conclusion

A robust ferroptosis-related prognostic signature was developed and validated with five independent datasets. Different tumor immune microenvironments were observed between the low-risk and high-risk groups. This signature may serve as a potential prognostic biomarker for LUAD in the future.

## Data Availability Statement

Publicly available datasets were analyzed in this study. This data can be found here: the datasets collected in the current study are available in the TCGA (https://portal.gdc.cancer.gov/) and GEO repository (https://www.ncbi.nlm.nih.gov/geo/).

## Author Contributions

YG and JH directed and designed the study. ZY, JW, RL, and HT extracted the data. SS and YY conducted the data analysis and experiments. SS wrote the manuscript. FT and QX reviewed and edited the manuscript. All authors read and approved the manuscript.

## Conflict of Interest

The authors declare that the research was conducted in the absence of any commercial or financial relationships that could be construed as a potential conflict of interest.

## Publisher’s Note

All claims expressed in this article are solely those of the authors and do not necessarily represent those of their affiliated organizations, or those of the publisher, the editors and the reviewers. Any product that may be evaluated in this article, or claim that may be made by its manufacturer, is not guaranteed or endorsed by the publisher.

## Footnotes

1
https://portal.gdc.cancer.gov

2
http://www.zhounan.org/ferrdb

3
https://tcia.at/home

4
http://www.cbioportal.org/

## Supplementary Material

Supplementary Table 1 Quantitative RT-PCR primers used in this study.

Supplementary Table 2 The list of 56 genes for model construction.

Supplementary Table 3 The significance of differences in infiltrated immune cell between risk groups.

Supplementary Table 4 List of significance for all 138 compounds.
