# Supplementary material for: Ferroptosis Characterization in Lung Adenocarcinomas Reveals Prognostic Signature With Immunotherapeutic Implication
Source: Front Cell Dev Biol. 2021 Oct 20;9:743724. doi: 10.3389/fcell.2021.743724 (PMC8563998; doi:10.3389/fcell.2021.743724)
Supplement: Supplementary Table 1 — Quantitative RT-PCR primers used in this study. [file Table_1.DOCX]

| **Supplementary Table 1丨**Quantitative RT-PCR primers. | | |
| --- | --- | --- |
| **Gene** | **Sequence** | **Length (nt)** |
| *ACTB-F* | GAGATCACTGCCCTGGCACC | 162 |
| *ACTB-R* | GATGGAGGGGCCGGACTCG |  |
| *EIF5A-F* | GGACTTCGAGACAGGAGATGC | 249 |
| *EIF5A-R* | TCATTCCTTTTGATGTTGGGGAC |  |
| *CACYBP-F* | CTCTGTGGAAGGCAGTTCAAA | 92 |
| *CACYBP-R* | TCAGGTAATCCCACCTTGTGTT |  |
| *CYCS-F* | GCCAGCGACTAAAAAGAGAATTA | 82 |
| *CYCS-R* | TGGCACTGGGAACACTTCAT |  |
| *ANLN-F* | TGCCAGGCGAGAGAATCTTC | 78 |
| *ANLN-R* | CGCTTAGCATGAGTCATAGACCT |  |
| *ARNTL2-F* | TGGCGCGTAAACTGGACAAA | 187 |
| *ARNTL2-R* | CCTCTTTCACATCCAACCACAAA |  |
| *PPM1M-F* | CTTGGTGCGGAGAGATGAGAT | 98 |
| *PPM1M-R* | GCTCAGGATAGACAAAGGCCAG |  |
